# Interval-Specific Congenic Animals for High-Resolution Quantitative Trait Loci Mapping

**Published:** 2008

**Authors:** Deaunne L. Denmark, Lauren C. Milner, Kari J. Buck

**Keywords:** Genetic theory of alcohol and other drug use, genetics and heredity, behavioral phenotypes, genetic trait, quantitative traits, quantitative trait locus (QTL), QTL mapping, congenics, interval-specific congenics (ISCs), congenic strains, animal models

Behavioral phenotypes (e.g., drug responses and withdrawal) are typically quantitative traits—characteristics that differ along a spectrum in the extent to which an individual possesses that characteristic. Such traits are determined by multiple genes, as well as by environmental factors and interactions among genes and environmental factors. The chromosomal regions containing these genes are commonly referred to as quantitative trait loci (QTLs). As described in the preceding article by Hitzemann and colleagues (pp. 270–271), researchers have developed a variety of strategies to attain greater precision when mapping QTLs (Darvasi 1998; Talbot et al. 1999), which is necessary for unbiased genome-wide approaches such as QTL mapping to be successful in ultimately identifying which gene(s) underlies a QTL’s phenotypic influence. Among these, some approaches are clearly superior for fine mapping QTLs associated with behavioral traits. One such strategy employs specially bred animals known as interval-specific congenics (ISCs) (sometimes called small donor segment congenics). This article introduces the use of these animals in mapping QTLs associated with certain responses to alcohol.

## What are Congenic and ISC Animals?

Classical congenic strains typically are derived from two inbred progenitor strains (i.e., a donor strain and a recipient, or background, strain) that differ substantially in one or more behaviors of interest. The two strains are bred to generate offspring (i.e., the F_1_ generation), which are intercrossed to generate second-generation (F_2_) progeny. Those F_2_ progeny that contain donor DNA in the chromosomal region of interest (identified using marker-assisted methods) are backcrossed to animals of the background strain. Over several backcross generations, donor strain DNA is selected for and retained within the region of interest, but is diluted elsewhere in the genome until, in the finished congenic, the donor strain sequence comprises less than 2 percent of the genetic “background.” In the majority of classical congenic strains, the donor congenic interval is too large (∼50 megabases [Mb] or more) to be easily or thoroughly analyzed with available techniques and to confidently define an individual QTL or gene of interest. The imprecision with which QTLs are initially mapped and isolated has hindered progress toward identification of their specific underlying gene(s) and the rigorous assessment of their potential pleiotropic effects (i.e., influence on multiple quantitative traits). These problems are alleviated by generating a series (or panel) of ISC lines/strains that contain small, partially overlapping donor intervals which together span the entire starting chromosomal region of interest. By determining which ISCs demonstrate QTL “capture” (i.e., phenotypic expression of the quantitative trait) and which do not, the QTL interval can be confidently narrowed to 1 to 2 Mb or less and further studied to, ideally, identify the specific causative gene(s).

The starting point for creating an ISC panel typically is an established congenic (or consomic^1^) strain in which a large chromosomal interval (or entire chromosome) from a donor strain has been stably introduced into an inbred background strain. Animals from the starting congenic strain are backcrossed to background strain animals to create first-generation offspring (N_1_), which are then backcrossed to the background strain. Some of these progeny will be recombinant within the starting interval of interest, resulting from recombination that occurs naturally in each generation during the formation of sperm and egg cells. Recombinant animals are identified using informative genetic markers within or flanking the QTL region to precisely define the proximal and distal boundaries of the donor segment. Individual recombinants containing a desired interval again can be backcrossed to background strain animals, resulting in multiple offspring that all carry the same small congenic interval; this is known as an ISC line (ISCL). At the same time that the ISCL is generated, and in subsequent backcross generations, additional recombinants are sought in order to further narrow the interval of interest. In ISCL animals, the desired donor interval is found only in one copy of the respective chromosome, as the second copy is always inherited from the background strain. To obtain animals that carry the donor piece on both chromosomes (i.e., donor region homozygotes), ISCL mice are simply intercrossed. One-quarter of the intercross progeny will inherit the donor segment from both parents and constitute an ISC strain (ISCS). The entire process requires only three generations beyond the starting congenic/consomic strain to develop the ISCLs and, if desired, two additional generations to establish the finished ISCSs.

**Figure 16:**
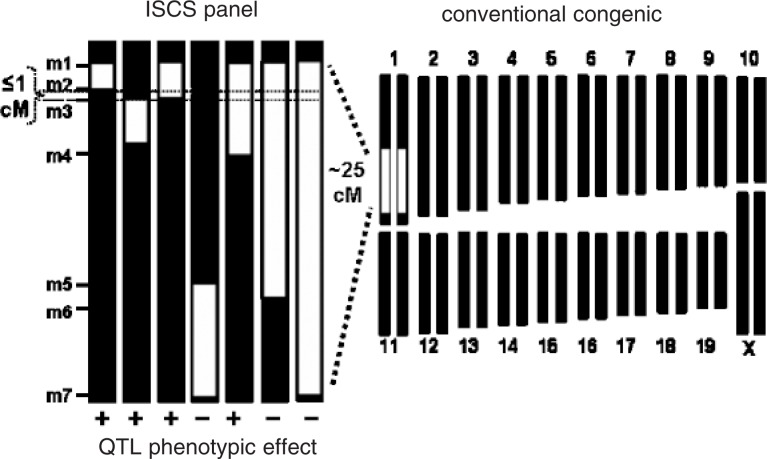
General scheme for production of interval-specific congenic (ISC) animals and fine mapping of quantitative trait loci (QTLs). ISCs are most efficiently and cost-effectively generated from an already-established congenic strain, which typically contains a large, QTL-containing interval of donor strain DNA (white) on one of the chromosomes (here, chromosome 1) of the recipient, or background strain (black). By repeatedly backcrossing this congenic strain with the background strain and selecting appropriate offspring based on informative genetic markers (m1 to m7) and the characteristic (i.e., phenotype) of interest, one can identify the smallest chromosomal interval necessary to capture the QTL phenotype, often achieving a resolution of 1centiMorgan (cM) or less (small dotted lines).

As stated above, the determination of which ISCs demonstrate “capture” by behavioral or physiological testing can confidently narrow a QTL interval to just a fraction of the original starting size (see figure 16). Importantly, with an appropriate design, even a small ISC panel can provide definitive QTL mapping to a clearly delimited 1 to 2 Mb interval (Fehr et al. 2002; Shirley et al. 2004; Kozell et al. 2008). This represents a substantial improvement over standard mapping strategies, which yield only statistically based confidence intervals on the order of 10 to 30 centiMorgans (cM), corresponding to about 20 to 50 Mb in mice (Glazier et al. 2002). ISCLs are particularly efficient when only one copy of a gene is sufficient for eliciting the desired effects (i.e., for dominant effects). ISCSs offer the advantages of requiring fewer animals for additive effects, and the ability to finely map QTLs of modest effect size on the trait under investigation. With both strategies, a QTL can be evaluated in relative genetic isolation from other contributing genes on a uniform (inbred) genetic background.

For quantitative traits determined by multiple genes, this approach allows researchers to dissect the polygenic effects because each ISCS carries only one specific QTL-containing region (Darvasi 1997). Moreover, because all animals in an ISCS have an identical genotype, random changes in the frequency of specific gene variants in the population—a process known as genetic drift—are avoided. As a result, ISC animals are becoming increasingly appreciated as one of the most robust tools available for novel discovery of the genes underlying alcohol-related traits. The approach is most commonly used with mice, which have a relatively short generation cycle and for whom a large number of genetic markers has been identified. Moreover, many mouse congenics/consomics already exist (see www.ohsu.edu/parc/animal_models.htm and www.genetics.ucla.edu/GTM) that can be used as starting points to develop unique ISC panels. Additionally, with rapidly expanding genomic information available for dozens of animal models, as well as an increasing number of established strains that can be used as a starting point, this approach is already proving similarly invaluable for researchers using rats (Carr et al. 2006; Radcliffe et al. 2006) and may soon be applicable to other species that also effectively model drug responses, as well as other complex traits.

## What Type of Information Can ISC Animals Provide?

In general, there are two primary ways in which ISC animals are informative, depending on the size of the original QTL, its genetic composition, and the extent of interval overlap between the different strains in a panel. In the most straightforward scenario, a QTL can be distilled to a single gene, as exemplified by the identification of a gene called *Mpdz* as underlying a QTL on mouse chromosome 4 associated with alcohol and barbiturate withdrawal (Shirley et al. 2004). In this case, the initial QTL interval spanned more than 40 cM, or approximately 80 Mb. Aggregate phenotypic analyses of six ISCLs reduced the QTL interval to only 1.8 Mb (Fehr et al. 2002; Shirley et al. 2004). Moreover, relatively few genes reside within this fine-mapped interval, further facilitating nomination of a single candidate. Subsequent molecular and haplotype analyses identified *Mpdz* as the only gene within the interval for which the presence of sequence and expression differences matched the observed withdrawal behavior, and which could therefore potentially explain the QTL effect on sedative-hypnotic drug withdrawal (Shirley et al. 2004).

This example highlights the power of the ISC approach to identify the influence of genes whose effect on drug response was previously unknown. However, even with fine mapping to 1 to 2 Mb or less, such a QTL interval may harbor more than one viable candidate. If that is the case, comparison of ISC and background strain animals with respect to potential variation in candidate gene expression, DNA sequence, or regulation can become important for confidently nominating those candidates that are the most promising. An example of this scenario is an alcohol withdrawal QTL on distal mouse chromosome 1 that has recently been fine mapped using the ISC strategy. In this case, behavioral analyses of a panel of four ISCSs reduced the QTL interval from more than 100 Mb to just 1.1 Mb (Kozell et al. 2008). However, the extraordinary gene density and variability in DNA sequence (i.e., polymorphism) of the region made identification of a single or few candidates particularly challenging; researchers found that 40 genes reside within the 1.1 Mb interval, of which 17 showed strain-specific expression and/or differences in coding DNA predicted to change the amino acid sequence of the resulting protein (Denmark and Buck 2008).

## Conclusions

ISC animals have been used successfully to precisely map multiple drug-response QTLs to 1 to 2 Mb intervals, resolve linked QTLs, and pare down the number of candidate genes in a given QTL region to a number amenable to thorough unbiased molecular analyses. Although ISC analysis is a tool with relatively high yield-to-cost ratio, significant resources still are required so that it can be applied most efficiently to genomic regions in which several QTLs are detected (see Table). In this context, ISC animals may be particularly useful when researchers want to assess QTLs that affect multiple behaviors (i.e., have pleiotropic effects). For example, QTLs on distal chromosome 1 are associated not only with acute and chronic alcohol and barbiturate withdrawal (Kozell et al. 2008; Buck et al 1999), but also influence sedative-hypnotic drug-conditioned aversion (Risinger and Cunningham 1998), sedative-hypnotic preference (Tarantino et al. 1998), and sedative-hypnotic drug-induced locomotor activation (Demarest et al. 1999) and hypothermia (Crabbe et al. 1994) in mice. This accumulation of drug response QTLs in one chromosomal region makes it tempting to speculate that the gene(s) associated with sedative-hypnotic drug withdrawal may have effects on additional behavioral responses and would therefore be an even more important target for further investigations. In this case, the use of ISC animals to compare additional drug responses with background strains, and whether the patterns for these differences match those for sedative-hypnotic withdrawal, can be extremely useful to obtaining more definitive confirmation of pleiotropy.

## Figures and Tables

**Table:** Alcohol Phenotypes Tested in Interval-Specific Congenic (ISC) Animals

**Chromosome**	**ISC (Background.Donor)**	**Species**	**Alcohol-Relevant Phenotype Tested**	**Reference/Group**
1	ISS.ILS; ILS.ISS	Mouse	Alcohol-induced loss of righting reflex	Bennett et al. 2002, 2008
	B6.D2; D2.B6	Mouse	Alcohol withdrawal seizure severity(acute & chronic)	Fehr et al. 2002; Shirley et al. 2004; Kozell et al. 2008
			Alcohol-conditioned taste aversion	C.L. Cunningham, unpublished data

2	ISS.ILS; ILS.ISS	Mouse	Alcohol-induced loss of righting reflex	Bennett et al. 2002; 2008
	B6.D2		Alcohol withdrawal seizure severity (acute & chronic)	J.C. Crabbe, unpublished data

4	D2.B6	Mouse	Alcohol withdrawal seizure severity (acute & chronic)	Fehr et al. 2002
			Alcohol withdrawal-associated depression-like behavior (tail suspension test)	K.J. Buck, unpublished data
			Homecage locomotor activity during alcohol withdrawal	K.J. Buck, unpublished data
	iP, iNP; iNP.iP	Rat	Alcohol preference (10 percent; 2-bottle choice with water)	Carr et al. 2006

5	ILS.ILS; ILS.ILS	Mouse	Alcohol-induced loss of righting reflex	Bennett et al., 2002; 2008

9	B6.D2	Mouse	Alcohol drinking (3 percent and 10 percent; 2-bottle choice with water)	T.J. Phillips, unpublished data
			Alcohol metabolism	J.C. Crabbe, unpublished data
			Drinking in dark	J.C. Crabbe, unpublished data
			Dopamine mimetic sensitivity and neuroadaptation	K.J. Buck, unpublished data

11	ISS.ILS; ILS.ISS	Mouse	Alcohol-induced loss of righting reflex	Bennett et al. 2002, 2008
	D2.B6	Mouse	Alcohol withdrawal seizure severity (acute)	Hood et al. 2006

15	ISS.ILS; ILS.ISS	Mouse	Alcohol-induced loss of righting reflex	Bennett et al. 2002, 2008 A. Janowsky & K.J. Buck unpublished data

19	D2.B6	Mouse	Dopamine transporter density	A. Janowsky & K.J. Buck Unpublished data
			Alcohol withdrawal seizure severity (acute & chronic)	K.J. Buck, unpublished data
			Dopamine mimetic sensitivity and neuroadaptation	K.J. Buck, unpublished data
			GBR-2935-induced locomotor activity	T.J. Phillips, unpublished data

NOTE: B6 = C57BL/6 mice; D2 = DBA/2 mice; iNP = inbred alcohol-nonpreferring rats; iP = inbred alcohol-preferring rats; ILS = inbred long-sleep mice; ISS = inbred short-sleep mice.

SOURCE: Bennett, B.; Beeson, M.; Gordon, L.; and Johnson, T.E. Reciprocal congenics defining individual quantitative trait loci for sedative/hypnotic sensitivity to alcohol. *Alcoholism: Clinical and Experimental Research* 26(2):149–157, 2002. PMID: 11964553 Bennett, B.; Carsone-Link, P.; Beeson, M.; et al. Genetic dissection of quantitative trait locus for ethanol sensitivity in long- and short-sleep mice. *Genes Brain and Behavior* 7(6):659–668, 2008. PMID: 18363857 Carr, L.G.; Habegger, K.; Spence, J.P.; et al. Development of congenic rat strains for alcohol consumption derived from the alcohol-preferring and nonpreferring rats. *Behavior Genetics* 36:285–90, 2006. PMID: 16470346 Fehr, C.; Shirley, R.L.; Belknap, J.K.; et al. Congenic mapping of alcohol and pentobarbital withdrawal liability loci to a <1 centimorgan interval of murine chromosome 4: Identification of Mpdz as a candidate gene. *Journal of Neuroscience* 22:3730–3738, 2002. PMID: 11978849 Hood, H.M.; Metten, P.; Crabbe, J.C.; and Buck, K.J. Fine mapping of a sedative-hypnotic drug withdrawal locus on mouse chromosome 11. *Genes Brain and Behavior* 5(1):1–10, 2006 PMID: 16436183

## Footnotes

1 In a consomic strain, one entire chromosome from the donor strain is retained among the recipient chromosomes.
